# Impact of untreated diabetes and COVID-19-related diabetes on severe COVID-19

**DOI:** 10.1016/j.heliyon.2022.e08801

**Published:** 2022-01-21

**Authors:** Emi Ushigome, Masahide Hamaguchi, Kazuki Sudo, Nobuko Kitagawa, Yuriko Kondo, Dan Imai, Tomohito Hattori, Takaaki Matsui, Masahiro Yamazaki, Teiji Sawa, Michiaki Fukui

**Affiliations:** a Department of Endocrinology and Metabolism, Graduate School of Medical Science, Kyoto Prefectural University of Medicine, Kyoto 602-8566, Japan; b Department of Anesthesiology, Graduate School of Medical Science, Kyoto Prefectural University of Medicine, Kyoto 602-8566, Japan

**Keywords:** COVID-19-related diabetes, Severe COVID-19, Untreated diabetes, COVID-19 comorbidity

## Abstract

Diabetes is a common comorbidity in patients with coronavirus disease (COVID-19) and contributes significantly to COVID-19 severity. We aimed to investigate the association between diabetic status and severe COVID-19. This prospective study included all COVID-19 patients admitted to our hospital, who were divided into four groups according to their diabetic status: no diabetes, treated diabetes, untreated diabetes, and COVID-19-related diabetes. Severe COVID-19 was defined as a condition that required the use of a ventilator. Of the 114 patients included in this study, 26 had severe COVID-19. The adjusted odds ratio (OR; 95% confidence interval [CI]) for severe COVID-19 was significantly higher in the treated diabetes, untreated diabetes, and COVID-19-related diabetes groups than in the no diabetes group (OR: 5.9, 95% CI [1.2–27.9]; OR 12.6, 95% CI [1.8–86.4]; and OR: 9.3, 95% [1.1–81.4], respectively). Findings from this study showed that the risk of severe COVID-19 was increased in treated diabetes, untreated diabetes, and COVID-19-related diabetes compared to no diabetes. Furthermore, the OR for severe COVID-19 was greater in untreated diabetes and COVID-19-related diabetes than in treated diabetes.

## 1 Introduction

Coronavirus disease (COVID-19), caused by severe acute respiratory syndrome coronavirus 2 (SARS-CoV-2), has caused more than 227 million infections and 4.6 million deaths worldwide since the outbreak began in December 2019, making it a global emergency (Medical News Today News Team, 2021; Zhu et al., 2020). Although the overall mortality rate is low, comorbid diabetes, especially type 2 diabetes, in patients with COVID-19 has reportedly been associated with a poor prognosis (Grasselli et al., 2020; Wu et al., 2020; Zhou et al., 2020). Moreover, higher mortality rates and adverse events were reported in patients with COVID-19-related diabetes, in which patients who did not have diabetes developed it due to COVID-19 infection or treatment, than in the non-diabetic population (Shrestha et al., 2021). Thus far, no studies have examined the association between diabetic treatment status and how it contributes to the severity of COVID-19. In this study, we aimed to determine whether treated, untreated, or COVID-19-related diabetes is associated with severe COVID-19.

## 2 Research design and methods

### 2.1 Study design

This prospective cohort study included all consecutive adult patients from March 4, 2020 to February 22, 2021 who had confirmed novel coronavirus–infected pneumonia (NCIP) and were admitted to Kyoto Prefectural University of Medicine Hospital, which was one of 55 publicly designated medical institutions for Class 1 infectious diseases in Japan and the only one in Kyoto, and mainly patients with COVID-19, who are expected to be severely ill, were admitted. We collected follow-up data until March 31, 2021. The inclusion criteria for the patients were as follows: patients aged over 20 years, inpatients, and those with a confirmed diagnosis of COVID-19. All procedures were approved by the local Research Ethics Committee of Kyoto Prefectural University of Medicine (ERB-C-1810-2) and were conducted in accordance with the Declaration of Helsinki. Written informed consent was obtained from all the patients.

### 2.2 Data collection

Admission blood samples were taken for biochemical measurements at the time of study entry. Each patient's characteristics, including age, sex, body mass index, smoking status, and history of hypertension, kidney disease, respiratory disease and cardiovascular disease were obtained from the initial questionnaire survey.

### 2.3 Definitions

In this study, the patients were divided into four groups according to their diabetes status: 1) no diabetes was defined as no history of diabetes, no increase in blood glucose levels during hospitalization (fasting blood glucose levels less than 126 mg/dL (7 mmol/L), and casual blood glucose levels less than 200 mg/dL (11.1 mmol/L)), and HbA1c levels of less than 6.5% on admission; 2) treated diabetes was defined as having a diagnosis of diabetes prior to admission and being treated in a medical institution; 3) untreated diabetes was defined as diabetes diagnosed prior to admission but untreated or interrupted, or that diagnosed for the first time at the time of admission; and 4) COVID-19-related diabetes was defined as diabetes that developed after hospitalization for COVID-19. The diagnosis of diabetes was based on the American Diabetes Association criteria (American Diabetes Association, 2021). Severe COVID-19 was defined as the need for a ventilator and/or extracorporeal membrane oxygenation management (Ministry of Health, Labour, and Welfare, 2021). Patients were defined as having renal disease if they had been diagnosed with glomerulonephritis, nephrotic syndrome, chronic kidney disease, chronic renal failure, or autosomal dominant polycystic kidney disease before admission, respiratory disease if they had been diagnosed with lung cancer, chronic obstructive pulmonary disease, interstitial pneumonia, emphysema, asthma, or pulmonary tuberculosis before admission, and cardiovascular disease if they had been diagnosed with myocardial infarction, angina pectoris, valvular disease, and cerebral infarction.

### 2.4 Statistical analysis

The baseline characteristics were summarized according to the four groups using the medians with ranges or numbers with percentages. We applied the Kruskal–Wallis test for continuous variables and the chi-squared test for categorical variables. We performed a logistic regression analysis to determine the relationship between diabetes status and severe COVID-19. In addition to the unadjusted model (Model 1), we set up multivariate models adjusted for COVID-19 severity risk factors such as age, sex, body mass index, hypertension, kidney disease, respiratory disease, cardiovascular disease and smoking status (Model 2), and adjusted for use of glucocorticoids, duration of COVID-19 incidence before hospitalization, and use of dialysis in addition to the variables in Model 2 (Model 3). The unadjusted and adjusted odds ratios (ORs) and 95% confidence intervals (CIs) were estimated. The SPSS software (version 19.0J, IBM Corp., Armonk, NY, USA) was used for statistical analyses, and a two-sided *P* value < 0.05 was considered statistically significant.

## 3 Results

During the follow-up period of this prospective study, there were 121 hospitalizations with confirmed NCIP in our hospital. Of these, 2 were under 20 years of age and 5 were unable to give consent to participate in the study, resulting in a final total of 114 patients. The clinical characteristics of the four groups at baseline are shown in Table 1. The median age and HbA1c were 65.0 years (interquartile range [IQR]: 50.0–73.3) and 6.1% (IQR: 5.7–7.0) (43.0 mmol/mol [IQR: 39.0–53.0]), respectively, and 78 patients (68.4%) were male. The proportions of patients in the no diabetes, treated diabetes, untreated diabetes, and COVID-19-related diabetes groups were 53.5%, 23.7%, 13.2%, and 9.6%, respectively. Before admission, 72% of the treated diabetes group received a dipeptidyl peptidase-4 inhibitor, 40% metformin, 8% pioglitazone, 44% sodium glucose transporter 2 inhibitors, 4% glucagon-like peptide 1 analog, and 12% insulin. Twenty-six patients (22.8%) became critically ill. The unadjusted ORs (95% CI) for severe COVID-19 in the treated diabetes, untreated diabetes, or COVID-19-related diabetes groups were significantly higher than that in the no diabetes group (OR: 11.3, 95% CI [2.8–46.1]; OR: 16.9, 95% CI [3.6–79.0]; and OR: 23.2, 95% CI [4.4–122.0], respectively) (Table 2). The adjusted ORs (95% CI) for severe COVID-19 in the treated diabetes, untreated diabetes, and COVID-19-related diabetes groups were significantly higher than that in the no diabetes group (OR: 5.9, 95% CI [1.2–27.9]; OR 12.6, 95% CI [1.8–86.4]; and OR: 9.3, 95% [1.1–81.4], respectively) (Table 2).

**Table 1:** Patient characteristics.

	All (114)	Non-diabetes (61)	Treated diabetes (27)	Untreated diabetes (15)	COVID19-related diabetes (11)	P value
Male	78 (68.4)	34 (55.7)	20 (74.1)	13 (86.7)	11 (100.0)	0.006
Age, years	65.0 (50.0–73.3)	56.0 (41.5–74.5)	70.0 (58.0–74.0)	68.0 (52.0–72.0)	72.0 (66.0–76.0)	0.012
Body mass index, kg/m^2^	23.4 (20.4–25.8)	22.2 (20.0–25.1)	25.2 (22.9–26.5)	24.8 (20.4–32.2)	23.3 (19.0–24.2)	0.018
Hemoglobin A1c, %	6.1 (5.7–7.0)	5.7 (5.5–6.0)	7.6 (6.5–8.1)	7.0 (6.7–7.7)	5.9 (5.8–6.4)	‹0.001
Hemoglobin A1c, mmol/mol	43.0 (39.0–53.0)	39.0 (37.0–42.0)	60.0 (48.0–65.0)	53.0 (50.0–61.0)	41.0 (40.0–46.0)	‹0.001
Use of glucocorticoids	55 (48.2)	19 (31.1)	17 (63.0)	9 (60.0)	10 (90.9)	‹0.001
Use of dialysis	6 (5.3)	1 (1.6)	3 (11.1)	1 (6.7)	1 (9.1)	0.279
Severe COVID-19	26 (22.8)	3 (4.9)	10 (37.0)	7 (46.7)	6 (54.5)	‹0.001
Duration of COVID-19 before hospitalization, days	7.0 (4.0–9.0)	6.0 (4.0–8.0)	7.0 (4.0–9.0)	7.0 (4.0–10.0)	11.0 (6.0–13.0)	0.060
Smoking status						
current	29 (26.9)	12 (21.1)	5 (19.2)	6 (40.0)	6 (54.5)	0.062
past	25 (21.4)	10 (17.5)	7 (25.9)	5 (33.3)	3 (27.3)	0.258
Hypertension	48 (42.1)	21 (34.4)	14 (51.9)	5 (33.3)	8 (72.7)	0.065
Kidney disease	17 (14.9)	8 (13.1)	5 (18.5)	0 (0.0)	4 (36.4)	0.070
Respiratory disease	17 (14.9)	7 (11.5)	3 (11.1)	6 (40.0)	1 (9.1)	0.035
Cardiovascular disease	23 (20.2)	8 (13.1)	7 (25.9)	2 (13.3)	6 (54.5)	0.012

Abbreviations: COVID-19, coronavirus disease 2019; Hemoglobin A1c, glycated hemoglobin.

Data are summarized by median (range) or number (%).

**Table 2:** Unadjusted and adjusted odds ratios for severe COVID-19.

	Model 1		Model 2^a^		Model 3^b^	
	Unadjusted odds ratio (95% CI)	P value	Adjusted odds ratio (95% CI)	P value	Adjusted odds ratio (95% CI)	P value
Non-diabetes	1		1		1	
Treated diabetes	11.3 (2.8–46.1)	0.001	7.4 (1.7–32.8)	0.008	5.9 (1.2–27.9)	0.025
Untreated diabetes	16.9 (3.6–79.0)	‹0.001	13.4 (2.3–79.4)	0.004	12.6 (1.8–86.4)	0.010
COVID-19-related diabetes	23.2 (4.4–122.0)	‹0.001	16.8 (2.2–129.6)	0.007	9.3 (1.1–81.4)	0.044

Abbreviations: CI, confidence interval; COVID-19, coronavirus disease 2019.

a Model 2: Odds ratios were adjusted for age, sex, body mass index, hypertension, kidney disease, respiratory disease, cardiovascular disease and smoking status.

b Model 3: Odds ratios were adjusted for variables in model 2 and additional adjustment for use of glucocorticoids, duration of COVID-19 incidence before hospitalization, and use of dialysis.

## 4 Discussion

This report, to the best of our knowledge, is the first to examine the relationship between diabetes status (i.e., treated or untreated) and severe COVID-19. Moreover, the OR for severe COVID-19 was greatest for COVID-19-related diabetes in this study. Currently, even though the underlying mechanisms linking comorbid diabetes and severe COVID-19 are unclear, several potential mechanisms of interaction have been identified. Hyperglycemia and insulin resistance affect metabolic abnormalities and the immune function of the cellular components of the innate immune system by releasing cortisol, catecholamines, cytokines, glucagon, and growth hormone (Wierusz-Wysocka et al., 1988). This impairment of the innate immune system due to hyperglycemia contributes to mortality and other severe diseases. Metabolic abnormalities can reduce the function of macrophages and lymphocytes, leading to altered cytokine profiles and impaired immune function (Xiu et al., 2014). For instance, it has been reported that diabetic COVID-19 patients had a more activated inflammatory response and suppressed immunity compared to nondiabetic COVID-19 patients (Guo et al., 2020). A cytokine storm has often been observed in COVID-19 deaths and is considered a major factor promoting disease progression. These findings suggest that COVID-19 patients with diabetes are more susceptible to overactive inflammation and imbalanced immune responses, which may be involved in the rapid deterioration of patients with COVID-19. Hyperglycemia and insulin resistance also impair the vascular endothelium and promote thrombus formation through oxidative stress, endothelial dysfunction, platelet hyperactivity, and inflammation (Kaur et al., 2018). Further, COVID-19 reportedly predisposes an individual to thrombotic diseases ranging from microvascular thrombosis to venous or arterial thrombosis (Bikdeli et al., 2020). Thus, the risk of thrombotic complications and death may be significantly increased in COVID-19 patients with diabetes.

In our study, the OR of severe COVID-19 was higher in the untreated diabetes group than in the treated diabetes group, even though HbA1c levels were comparable between the two groups. Although the present study did not reveal a causal relationship between untreated diabetes and severe COVID-19, there are several reports on antihypertensive agents that may be involved in this causal relationship. Rao et al. (2020) reported that diabetes might increase the expression of ACE2, which is a cellular receptor required for both SARS-CoV and SARS-CoV-2 infections (Zhang et al., 2020) in the lung. Several anti-diabetic medications reportedly affect the expression and activity of ACE2 (Pal and Bhadada, 2020). Pioglitazone has been shown to enhance ACE2 activity in the insulin-sensitive tissues of rats and to reduce A disintegrin and metalloproteinase-17 (ADAM17) activity in human skeletal muscles (Tripathy et al., 2013). In diabetic mice, insulin administration has been shown to weaken the function of renal ADAM17, which cleaves ACE2 (Salem et al., 2014). Similarly, a glucagon-like peptide 1 analog increases ACE2 expression in the heart and lungs of rats with type 1 diabetes (Romaní-Pérez et al., 2015). An increase in the ACE2/ACE ratio with sodium glucose transporter 2 inhibitors in the presence of ACE inhibitors has also been reported (Kawanami et al., 2017). Additionally, a meta-analysis has shown that the use of dipeptidyl peptidase-4 inhibitors is associated with reduced mortality in patients with COVID-19, with the association being weaker in patients receiving concomitant metformin and/or ACE inhibitors (Rakhmat et al., 2021). A systematic review has shown that the use of metformin may lead to improved clinical outcomes in patients with mild to moderate COVID-19, especially in women with diabetes (Zangiabadian et al., 2021). We investigated the association between the type of antidiabetic treatment received and the severity of COVID-19 in our study. In univariate analysis, metformin was negatively correlated with the severity of COVID-19, but the association disappeared in multivariate analysis.

In the present study, the OR of severe COVID-19 was higher in the COVID-19-related diabetes group than in the treated diabetes group; it is possible that the development of COVID-19-related diabetes may itself represent a COVID-19 disease state. Zangiabadian et al. reported that about half of the SARS-CoV-2 infected patients had elevated blood glucose levels (2021). In addition, pancreatic damage has been reported to occur in patients with severe COVID-19, suggesting that SARS-CoV-2 may bind to ACE2 in the pancreas and directly damage the islets, worsening glycemic control in COVID-19 patients (Liu et al., 2020). Koufakis et al. reported that sodium-glucose co-transporters (SGLT) 1 upregulation could result in increased intestinal glucose absorption and subsequently promote the development of hyperglycemia in COVID-19 (Koufakis et al., 2021). Furthermore, systemic insulin resistance and damage to the beta cells by activated innate immune cells are among the factors responsible for elevated blood glucose levels. The released inflammatory mediators directly inhibit the action of insulin and are involved in the development and progression of insulin resistance (Shoelson et al., 2006). In the present study, many severe cases had high inflammatory responses and required high doses of insulin (Ushigome et al., 2021). Importantly, treatment-induced increases in blood glucose levels also need to be considered. The results of a recent clinical study have reported that glucocorticoid therapy is effective in cases of severe COVID-19 (RECOVERY Collaborative Group et al., 2021). In the present study, more than half the patients were administered glucocorticoids. High-dose glucocorticoid therapy is associated with beta-cell dysfunction, insulin resistance, and leads to elevated blood glucose levels in patients with or without a history of diabetes (Ho et al., 2003). COVID-19-related diabetes may reflect the most severe condition in COVID-19, including pathogenesis and treatment.

There are some limitations to this study. First, the sample size was small, which limits the statistical power of the study. Second, in this study, no diabetes was defined based on the patient's self-report, in addition to no increase in blood glucose levels during hospitalization and HbA1c levels of less than 6.5% on admission. We did not search medical health records and may not be completely accurate. Additionally, it is uncertain whether the results of this study can be generalized to other ethnic groups (Buikema et al., 2021).

In conclusion, this study showed that the risk of severe COVID-19 was increased in treated diabetes, untreated diabetes, and COVID-19-related diabetes compared to no diabetes. Furthermore, the OR for severe COVID-19 was greater in untreated diabetes and COVID-19-related diabetes than in treated diabetes. This knowledge will help clinicians categorize patients early on and modify their treatment, accordingly, thereby improving the prognosis. It is also important to continue diabetic treatment to prevent future cases of severe COVID-19. Further observational studies need to be conducted to verify the results of this study.

## Declarations

### Author contribution statement

Emi Ushigome, Masahide Hamaguchi, Michiaki Fukui: Conceived and designed the experiments; Performed the experiments; Analyzed and interpreted the data; Wrote the paper.

Nobuko Kitagawa, Yuriko Kondo, Dan Imai, Tomohito Hattori, Takaaki Matsui, Masahiro Yamazaki: Conceived and designed the experiments; Performed the experiments; Wrote the paper.

Kazuki Sudo, Teiji Sawa: Performed the experiments; Wrote the paper.

### Funding statement

This research did not receive any specific grant from funding agencies in the public, commercial, or not-for-profit sectors.

### Data availability statement

Data will be made available on request.

### Declaration of interests statement

The authors declare no conflict of interest.

### Additional information

No additional information is available for this paper.
